# Refractory period in network models of excitable nodes: self-sustaining stable dynamics, extended scaling region and oscillatory behavior

**DOI:** 10.1038/s41598-017-07135-6

**Published:** 2017-08-02

**Authors:** S. Amin Moosavi, Afshin Montakhab, Alireza Valizadeh

**Affiliations:** 1 Department of Physics, College of Sciences, Shiraz University, Shiraz, 71946-84795 Iran; 2 Department of Physics, Institute for Advanced Studies in Basic sciences (IASBS), Zanjan, 45137-66731 Iran

## Abstract

Networks of excitable nodes have recently attracted much attention particularly in regards to neuronal dynamics, where criticality has been argued to be a fundamental property. Refractory behavior, which limits the excitability of neurons is thought to be an important dynamical property. We therefore consider a simple model of excitable nodes which is known to exhibit a transition to instability at a critical point (*λ* = 1), and introduce refractory period into its dynamics. We use mean-field analytical calculations as well as numerical simulations to calculate the activity dependent branching ratio that is useful to characterize the behavior of critical systems. We also define avalanches and calculate probability distribution of their size and duration. We find that in the presence of refractory period the dynamics stabilizes while various parameter regimes become accessible. A sub-critical regime with *λ* < 1.0, a standard critical behavior with exponents close to critical branching process for *λ* = 1, a regime with 1 < *λ* < 2 that exhibits an interesting scaling behavior, and an oscillating regime with *λ* > 2.0. We have therefore shown that refractory behavior leads to a wide range of scaling as well as periodic behavior which are relevant to real neuronal dynamics.

## Introduction

Various physical, biological and chemical systems are composed of interacting excitable agents and thus networks of excitable nodes are widely used to model the behavior of such systems. Examples of such systems include tectonic plates^1, 2^, Neural networks^3–7^, models of self-organized criticality (SOC)^8–11^, and epidemic (contagion) spreading^12–16^. Oftentimes excitable nodes are modeled with threshold dynamics as in the case of sandpile models of SOC which are thought to underlie the wide range of scale-invariant behavior seen in Nature.

Criticality in cortical dynamics is by now a widely studied and well established field^17^. Neuronal avalanches have been reported as collective scale-invariant behavior of neurons in the cortical layers of mammalian brain^3, 17–25^. Probability distribution functions of duration and size of the neuronal avalanches are power law functions observed in a wide range of space and time which are thought to be hallmarks of critical systems. In addition to avalanche statistics, branching ratio has also been used to characterize various time-series in order to establish critical dynamics of the brain^3^. Criticality of the brain is therefore the subject of a myriad of theoretical as well as experimental studies^26–34^. Critical dynamics is believed to underlie many functional advantages in a healthy brain, including learning^35^, optimal dynamic range^5, 36–39^, efficient information processing^32^, as well as optimal transmission and storage of information^4^.

Many excitable agents often display refractory behavior. This behavior which is characterized by a time scale (i.e. refractory period) is a time during which the excited agent cannot be re-excited. The presence of such refractory period can clearly affect the collective dynamics of excitable nodes. Neuronal systems are perfect examples of networks of excitable nodes with refractory period^40^.

A particularly useful model of excitable agents considers a random network of such nodes with quenched excitatory connection weights and probabilistic dynamics^41^. It is well-known that such a model exhibits a phase transition between stable and unstable regimes as the average weight of connections is increased^37, 38, 42^. More recently, it has been shown that the addition of inhibitory connections leads to a ceaseless dynamics which exhibits critical behavior by fine-tuning the system to the transition point associated with the mentioned instability^43^. This fine-tuning essentially leads to an intricate balance between excitatory and inhibitory connections which may not be *a priori* available and thus a non-generic behavior. Here, we propose to study the original model (without inhibitory connections) in the presence of refractory period. Interestingly, we find that refractory period leads to stable dynamics in the entire range of parameter. However, and perhaps more interestingly, we are able to identify a critical point with robust finite-size scaling behavior, a wide critical-like regime with interesting scaling behavior, as well as a regime with periodic behavior. Therefore, we show that refractory period in addition to stabilizing the dynamics leads to a wide range of parameters which show scaling or periodic behavior which are hallmarks of real neuronal dynamics.

This article is organized as follows: The following section discusses the model. Next, we show our analytical and numerical results, respectively. We close by providing concluding remarks.

## Model

The model consists of *N* excitable nodes on a random directed graph where every two nodes are connected with probability *q*. The average out-degree *and* in-degree of the network is equal to 〈*k*〉 = *qN*. Weights of the connections (*w*_*ij*_), that form the adjacency matrix of the network, are randomly chosen real numbers in the range of [0, 2*σ*] with the average connection weight of *σ*. If node *i* is not connected to node *j*, their connection weight is set to zero (*w*_*ij*_ = 0). Every node can be in one of active or quiescent states. Activity of the network is shown by the spatio-temporal binary variable, *A*_*i*_(*t*), i.e. if the node *i* is active at time *t* then *A*_*i*_(*t*) = 1 and when the node is quiescent *A*_*i*_(*t*) = 0. The probability of a node to be activated at time *t* + 1 is equal to

1 $$p({A}_{i}(t+\mathrm{1)}=\mathrm{1)}={\delta }_{\mathrm{0,}{A}_{i}(t)}f(\sum _{j=1}^{N}\,{w}_{ij}{A}_{j}(t))$$

where ${\delta }_{\mathrm{0,}{A}_{i}(t)}$ is the Kronecker delta, which is equal to zero (one) if *A*_*i*_(*t*) = 1 (0), implying refractory period of one time step, i.e. the activation probability of a node that is active at time *t* will be equal to zero at *t* + 1. *f* is a transfer function that transfers the total input of a node $({\sum }_{j=1}^{N}\,{w}_{ij}{A}_{j}(t))$ at time *t* to the activation probability of that node at time *t* + 1, and is chosen to be

2 $$f(y)=\{\begin{array}{ll}y, & 0\le y\le 1\\ 1, & y > 1.\end{array}$$

In this work, our focus is on the aggregate activity of the system which is defined as the number of active nodes at each time step and is equal to ${x}_{t}={\sum }_{i=1}^{N}\,{A}_{i}(t)$. We use *x*_*t*_ as a dynamical variable to analyze stability as well as statistical properties of the system.

Before presenting our results, it is instructive to remark on properties of the model without a refractory period. It is well known that the behavior of this model, without a refractory period, is governed by the largest eigenvalue of the adjacency matrix^37–39, 42^ which is equal to *λ* = *σ*〈*k*〉 for the random graph that we use^42, 44^. It has been shown that this system exhibits stability and instability in activity for *λ* ≤ 1 and *λ* > 1, respectively. In the case of stability, the system requires external drive to be activated and *x* = 0 is the stable attractor of the dynamics. When unstable (*λ* > 1) the activity of the system increases and saturates at *x* = *N*. The critical point of the system is *λ* = 1 where the system undergoes a transition from stability to instability. Poised at the critical point, the system exhibits scaling behavior for statistical properties of cascades of activity (avalanches) that start by an external perturbation. Our main goal in this paper is to scrutinize the behavior of the system when refractory period is introduced into the dynamics, i.e. delta function in Eq. (1). We are particularly interested in stability of dynamics and/or whether generic scaling behavior similar to cortical samples could arise in the model.

## Results

### Mean-field analysis

Dynamics of the system with refractory period is governed by Eq. (1). It is clear that the dynamics is strongly affected by the interaction weights (*w*_*ij*_). We can roughly explain the behavior of the system by considering different extremes of *λ* which is proportional to the average weight of connections *σ*. For small values of $\lambda \ll 1$ the interaction weights are small and any activity that starts by an initial perturbation is expected to die out very fast leading to a stability of the fixed point *x* = 0. In the other extreme, for large values of $\lambda \gg 1$, due to large values of interaction weights the probability of firing is expected to be equal to one for any node that receives input and is also quiescent at the time (see Eq. (1)). Therefore, at any given time, after a transient period, there are two sets of nodes: *x* that are active, and *N* − *x* that are in refractory period and will be active in next time step. Clearly, the system exhibits oscillations in this limit where all active nodes become quiescent and vice versa. We generally expect that there exists an important range of *λ* over which the system changes its behavior from a ceasing stable phase to a cease-less periodic phase. It is possible that the transition passes though a critical point or region.

In order to analyze the behavior of the model we use the aggregate activity of the system *x*_*t*_ as a function of time, and calculate the *activity dependent* branching ratio *b*(*M*)^45^ which is the expectation value of *x*_*t*+1_/*M* when there are *M* active nodes at time *t*,

3 $$b(M)=E(\frac{{x}_{t+1}}{M}|{x}_{t}=M).$$

It is clear from definition of *b* that for a given value of *x*_*t*_ = *M*, if *b*(*M*) > 1 (*b*(*M*) < 1) an average increase (decrease) in activity is expected in the next time step. Therefore, *b*(*M*) can provide important statistical information about the behavior of the system for the entire range of possible values of *x*_*t*_.

Galton-Watson theory of branching process holds that a branching ratio less, equal or larger than one respectively bespeaks sub-critical, critical and super-critical phases in a system^46^. But, the activity dependent branching ratio, due to its variability with respect to activity *x*, is different from the branching ratio defined in the Galton-Watson process and therefore provides much more information about the dynamics of a system. We thus consider criticality with regard to the activity dependent branching ratio. We define a system as critical if there exists a range *R* (*M*∈*R*) which is accessible by the long term dynamics of the system and exhibits two characteristics: (i) the value of the activity dependent branching ratio must be equal to one over *R* in the thermodynamic limit, i.e. ${\mathrm{lim}}_{N\to \infty }\,b(M\in R)=1$, and (ii) *R* must go to infinity as *N* → ∞. Condition (i) has to do with critical systems being (on the average) unpredictable. Condition (ii) has to do with lack of characteristic scale for a critical systems in the thermodynamic limit. We note that *b*(*M*) has been used to ascertain criticality in a wide range of systems including sandpile models of SOC or solar flares^45^ as well as neural networks^33^.

We begin by employing a mean-field approach in order to calculate the activity dependent branching ratio, analytically. If there are *M* active nodes at a time *t*, then at time *t* + 1 every node will receive an input with the weight of $\frac{M\langle k\rangle }{N}$, and if a node is quiescent at time *t* will be activated with probability of $f(\frac{M\langle k\rangle \sigma }{N})$. The largest eigenvalue of the connection matrix is *λ* = *σ*〈*k*〉 and the activation probability can thus be written as $f(\frac{M\lambda }{N})$. Since $\frac{M\lambda }{N}\ge 0$, two situations are possible: (a) $0\le \frac{M\lambda }{N}\le 1$: in this case $f(\frac{M\lambda }{N})=\frac{M\lambda }{N}$, and the probability of having exactly *x*_*t*+1_ = *z* active nodes at *t* + 1 when there are *x*_*t*_ = *M* active nodes at time *t* can be approximated as a binomial probability function, i.e. due to the refractory period of one time step, there are *N* − *M* nodes that can be activated with probability $\frac{M\lambda }{N}$. (b) $\frac{M\lambda }{N} > 1$: in this case $f(\frac{M\lambda }{N})=1$ and every quiescent node that receives input in a time step will be activated in the next time step. Therefore, the conditional probability is obtained as:

4 $$P({x}_{t+1}=z|{x}_{t}=M)=\{\begin{array}{ll}(\begin{array}{c}N-M\\ z\end{array})\,{(\frac{M\lambda }{N})}^{z}\,{(1-\frac{M\lambda }{N})}^{N-M-z} & \frac{M\lambda }{N} < 1\\ {\delta }_{z,N-M} & \frac{M\lambda }{N}\ge 1\end{array}$$

where the Kronecker delta indicates that all quiescent nodes will on the average be activated when *Mλ*/*N* > 1. We can therefore calculate the activity dependent branching ratio as:

5 $$b(M)=\frac{E({x}_{t+1}|{x}_{t}=M)}{M}=\{\begin{array}{ll}\lambda -\frac{\lambda }{N}M & M < \frac{N}{\lambda }\\ \frac{N}{M}-1 & M\ge \frac{N}{\lambda }\end{array}$$

**Figure 1:**
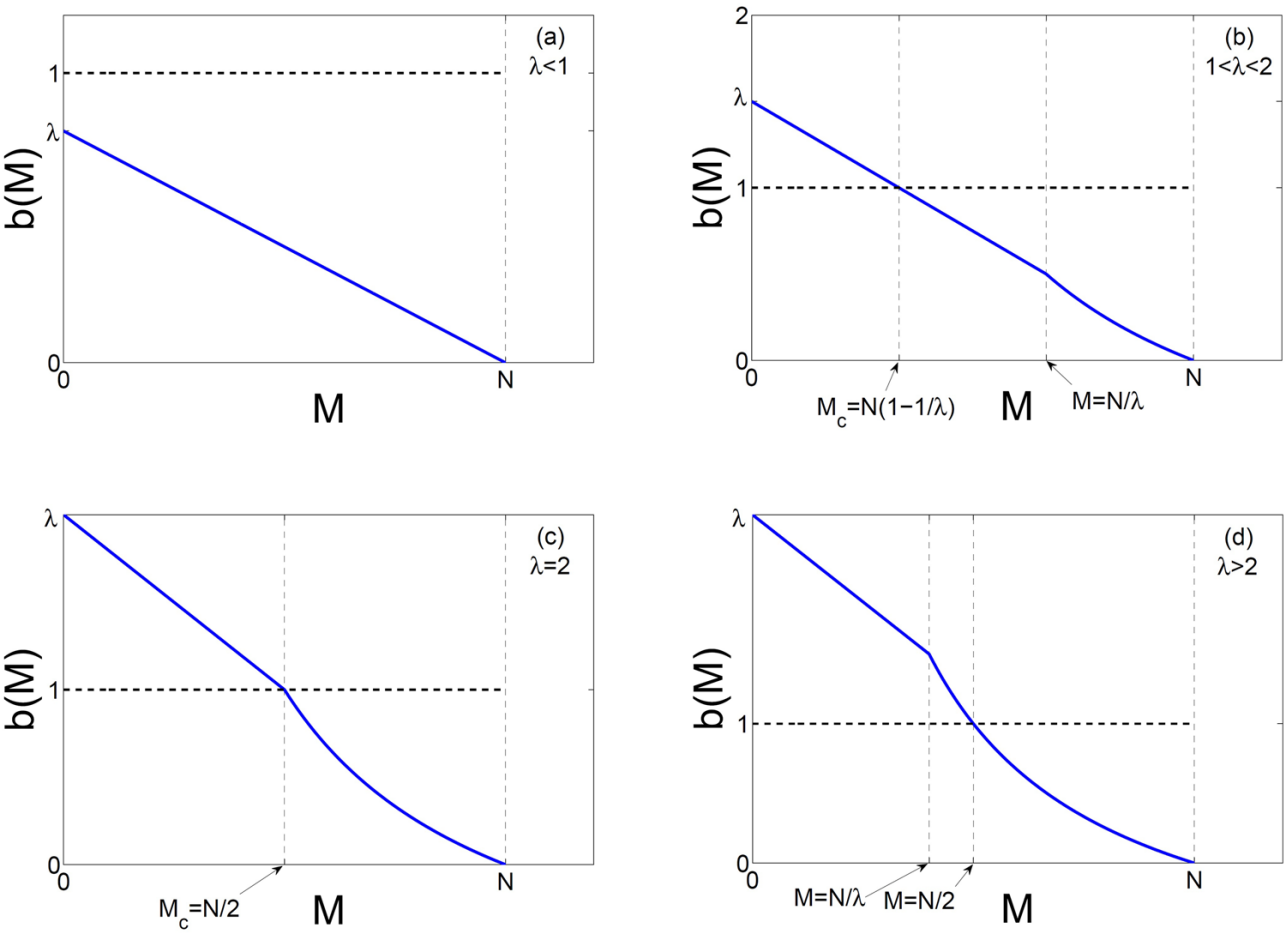
Activity dependent branching ratio for different parameter regimes of (**a**) *λ* < 1, (**b**) 1 < *λ* < 2, (**c**) *λ* = 2 and (**d**) *λ* > 2.

Having calculated the function *b*(*M*), we can present a mean-field analysis of the behavior of the system. As is clear from Eq. (5) the behavior crucially depends on the value of *λ*. Note that *b*(*M*) is a piecewise differentiable decreasing function of *M*, which has a linear as well as a nonlinear regime and goes to zero at *M* = *N*, see Fig. 1. For *λ* < 1 (see Fig. 1(a)), we have $M < \frac{N}{\lambda }$ and we are in the linear branch of $b(M)=\lambda -\frac{\lambda }{N}M$ which is always less than one. This indicates that the activity of the system will on average decrease until reaching the fixed point of *x*_*t*_ = x* = 0 for all initial perturbations. This is the stable sub-critical regime for which $b(M) < 1,\,\forall M$. Note that, when *λ* → 1, ${b(M)|}_{M={x}^{\ast }=0}\to 1$ and the stable fixed point is expected to exhibit critical behavior. Also note that in the *N* → ∞ limit, $b(M)=\lambda ,\,\forall M$ for *λ* ≤ 1 which indicates subcritical behavior for *λ* < 1 *and* critical behavior for *λ* = 1.

For 1 < *λ* < 2 (see Fig. 1(b)), the line *b*(*M*) = 1 will intersect *b*(*M*) in the linear regime at ${M}_{c}=N(1-\frac{1}{\lambda })$. Interestingly, due to the negative slope of *b*(*M*), x* = *M*_*c*_ will be the attractor of the dynamics as *M* < *M*_*c*_ (*M* > *M*_*c*_) *b*(*M*) will be larger (smaller) than one which would indicate increased (decreased) average activity until *x* = *M*_*c*_ is reached where *b*(*M*) = 1. This indicates a stable, ceaseless (${x}^{\ast }\ne 0$) dynamics which would exhibit critical behavior as *b*(*M* = *M*_*c*_) = 1. Note that for *λ* = 2 (see Fig. 1(c)), the critical attractor *M*_*c*_ will coincide with nonlinear regime of *b*(*M*) at ${M}_{c}=\frac{N}{2}$.

As *λ* is further increased, *λ* > 2 (see Fig. 1(d)) the line *b*(*M*) = 1 will intersect *b*(*M*) in the nonlinear regime and the simple analysis presented above will no longer hold. However, one can simply note that for $M > \frac{N}{\lambda }$, $E({x}_{t+1}|{x}_{t}=M)=N-M$ (see Eq. (5)) and $E({x}_{t+1}|{x}_{t}=N-M)=M$ which would indicate a period-2 oscillating behavior. For the case when *x*_*t*_ < *N*/2 (i.e. initial conditions in the linear regime), time evolution of the system will increase *x*_*t*_ as *b*(*M*) > 1 in this regime until we reach $M=\frac{N}{\lambda }$ after which the same periodic behavior would occur between $M=\frac{N}{\lambda }$ and $M=N-\frac{N}{\lambda }$. The fact that periodic behavior arises as a result of refractory period has previously been observed as in models of epidemic spreading^47, 48^. However, the case of refractory period larger than one presents an interesting case study, the details of which is presented in the Appendix.

**Figure 2:**
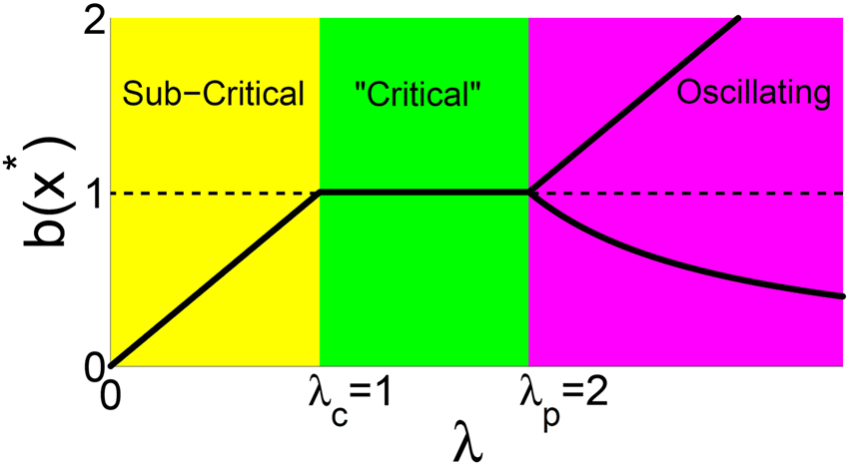
Activity dependent branching ratio evaluated at the attractors (*x* ^*^) of the dynamics as a function of *λ*. See text for details.

Figure 2 summarizes the mean-field behavior of the system. Three parameter regimes are presented. For 0 < *λ* < 1 the activity dependent branching ratio at the stable attractor of the dynamics is equal to *b*(x*) = *λ* < 1 and the system is sub critical. For 1.0 ≤ *λ* ≤ 2.0 the activity dependent branching ratio is *b*(x*) = 1.0 at the stable attractor of the dynamics and criticality is expected. For *λ* > 2 we have two values for the branching function and the dynamics jumps back and forth between these two values. *λ*_*c*_ = 1 and *λ*_*p*_ = 2 are the bifurcation points where the system changes behavior. while the value of *λ*_*c*_ is independent of refractory period, the value of *λ*_*p*_ (>1) depends on the choice of refractory period, see Appendix.

### Numerical calculation of b(M)

The above results portray the general behavior of the system in mean-field approximation where fluctuations have been ignored. However, as is well-known fluctuations are very important and tend to dominate system behavior in the critical regime. We therefore propose to study our system by extensive numerical simulations for *N* = 1 × 10^4^ up to 8 × 10^4^ and *q* = 0.01 in the range of 0.9 ≤ *λ* < 2 with particular focus on the critical behavior of the system. All activities are initiated by choosing a random site *i* and setting *A*_*i*_(*t* = 0) = 1 and following the ensuing dynamics according to Eqs (1 and 2). *x*_*t*_ is recorded for long times from which we can easily calculate *b*(*M*) numerically.

**Figure 3:**
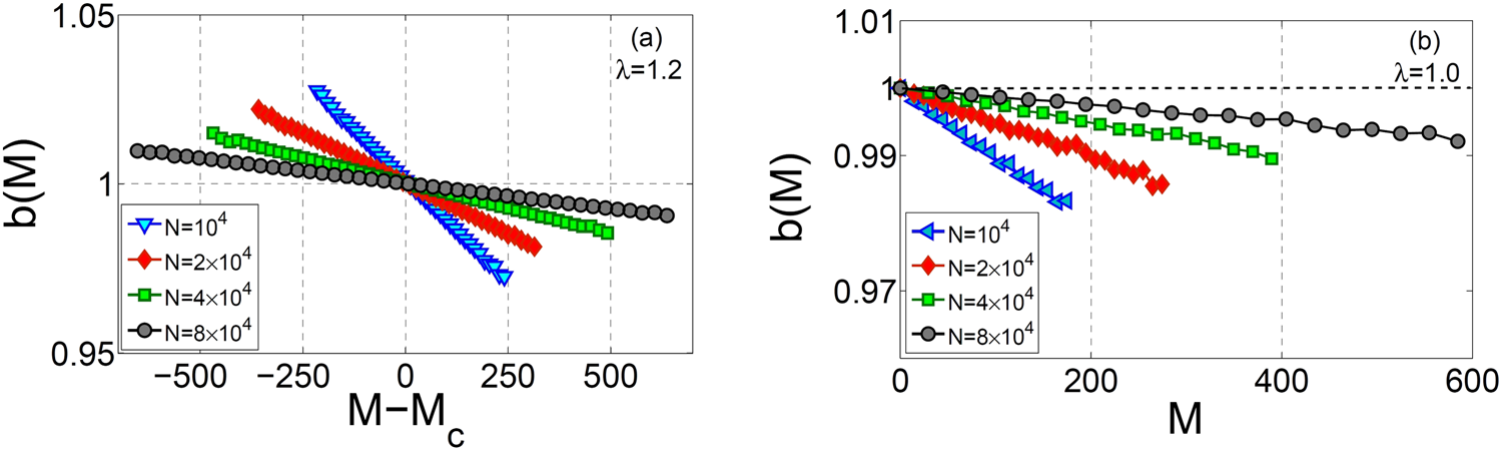
Activity dependent branching ratio for (**a**) *λ* = 1.2, (**b**) *λ* = 1 and different values of *N* = 10^4^, 2 × 10^4^, 4 × 10^4^, 8 × 10^4^.

Figure 3 shows our results for *b*(*M*) for various system sizes and *λ* = 1.2 as well as *λ* = 1.0, where criticality is expected. In both cases, our results clearly show a linear behavior in accordance with Eq. (5), where $b(M)=\lambda -\frac{\lambda }{N}M$ provides a prefect fit to the data. Note that as *N* is increased the range of system’s activity increases as the slope ($\tfrac{\lambda }{N}$) goes to zero. Therefore, one can expect that in the large system size limit *b*(*M*) → 1 for all accessible *M* indicating a critical behavior. We have also checked various other values of *λ* and have observed similar behavior to that of *λ* = 1.2 (above) for the range of 1 < *λ* < 2 (not shown).

### Avalanche statistics

**Figure 4:**
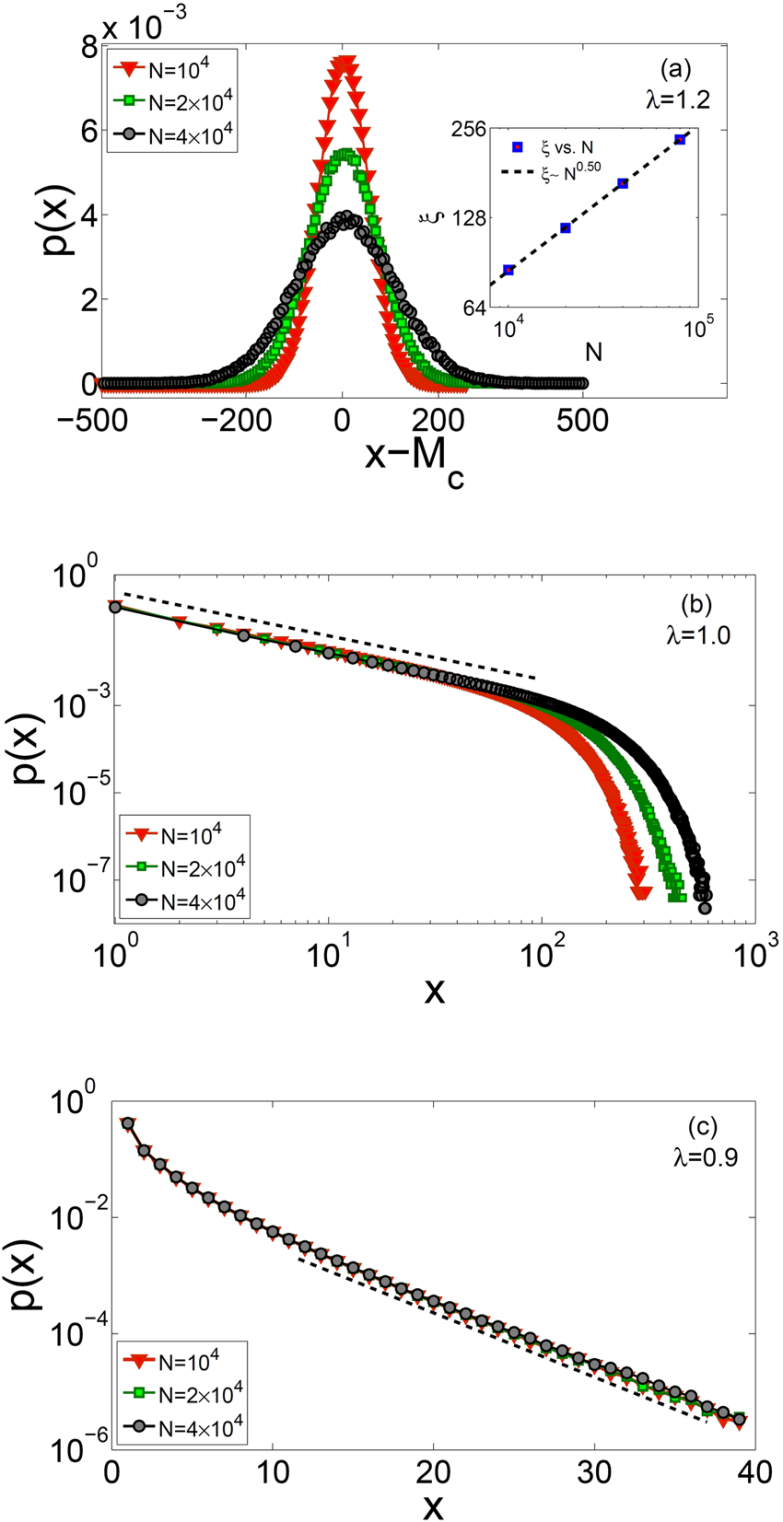
Probability distribution function of aggregate activity (*p*(*x*)) as a function of *x* for different values of *N*. Panel (a) is a linear plot showing a Gaussian function for *λ* = 1.2 with a maximum at *x* = *x*_*c*_ = *M*_*c*_. Inset is a log-log plot of the standard deviation *ξ* versus *N*. Panel (b) is a log-log plot showing power-law behavior for *λ* = 1.0, and panel (c) is a semi-log plot with *λ* = 0.9 showing exponential behavior.

In order to better understand the behavior of fluctuations about the attractors *x* ^*^, we have plotted the probability distribution function of system’s activity as *p*(*x*) in Fig. 4 for *λ* = 1.2, 1.0, and 0.9. For *λ* = 1.2 (Fig. 4(a)) we observe a Gaussian behavior which peaks exactly at $x={M}_{c}=N(1-\frac{1}{\lambda })$. It is interesting to note that our results indicate that the width of the Gaussian increases with the system size as $\xi \sim {N}^{0.5}$ (see inset of Fig. 4(a)), in accordance with the central limit theorem. For *λ* = 1.0 (Fig. 4(b)), we observe a distinctly different behavior as *p*(*x*) displays a power-law behavior with system size dependent cutoff. It is, however, important to note that *p*(*x*) is maximized at *x* = 0 as indicated by our mean-field analysis. In Fig. 4(c), we plot the same results for *λ* = 0.9 for various system sizes on a log-linear plot, all of which coincide on the same curve. We therefore conclude that *p*(*x*) displays an exponential behavior with a scale which is system size *independent*. Again, the attractor *x* = 0 appears as the most probable state, however, the size independent scale in (c) as opposed to size dependent scale in part (b) (and even (a)) is the distinction between sub-critical and critical systems.

**Figure 5:**
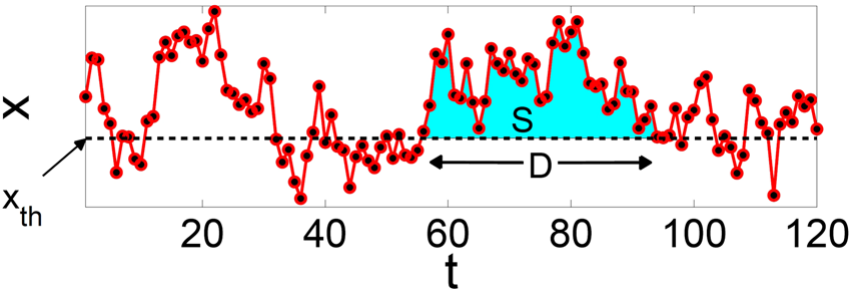
An avalanche is defined as excursion of the aggregate activity of nodes above a threshold value *x*_*th*_, *D* is the defined as the duration and the integral between *x*_*t*_ and *x*_*th*_ (the colored area) as the size (*S*) of the avalanche.

As we have defined a critical system based on the behavior of *b*(*M*) (see above), we have shown that our system exhibits critical behavior in the range 1 ≤ *λ* < 2. However, distinctly different behavior is observed for *P*(*x*) as *M*_*c*_ = 0 (Fig. 4(b)) changes to ${M}_{c}\ne 0$ (Fig. 4(a)). To better understand the critical behavior of the system we now turn our attention to avalanches. In the case of *λ* = 1.0 for which the stable attractor of the dynamics is x* = 0, the avalanches are well defined as the activity between two stabilities initiated by an external perturbation. For other values of 1 < *λ* < 2 over which the system exhibits self-sustaining behavior we define the avalanches (see Fig. 5), as the continuous aggregate activity of nodes above a threshold value *x*_*th*_. The number of time steps of an excursion above *x*_*th*_ is defined as duration (D) and the summation ${\sum }_{D}\,{x}_{t}-{x}_{th}$ as the size (*S*) of an avalanche.

Probability distribution function of size and duration of avalanches (*P*(*y*), $y\in \{S,D\}$) are calculated for systems with different *N* over the parameter regime 1 ≤ *λ* ≤ 2. In the case of criticality, these probability distribution functions are expected to exhibit power-law behavior with a cutoff which is an increasing function of *N*. The usual scaling ansatz for such a behavior is $P(y)\sim {y}^{-{\tau }_{y}}{g}_{y}(y/{N}^{{\beta }_{y}})$, where *g*_*y*_ is a universal cutoff function that is identical for different system sizes. *τ*_*y*_ is the critical exponent and *β*_*y*_ is referred to as the finite-size scaling exponent. When criticality holds, if we rescale $y\to y/{N}^{{\beta }_{y}}$ and $P(y)\to {y}^{{\tau }_{y}}P(y)$ then the plots of rescaled variables must collapse into one universal curve for the correct values of *τ*_*y*_ and *β*_*y*_^11^.

**Figure 6:**
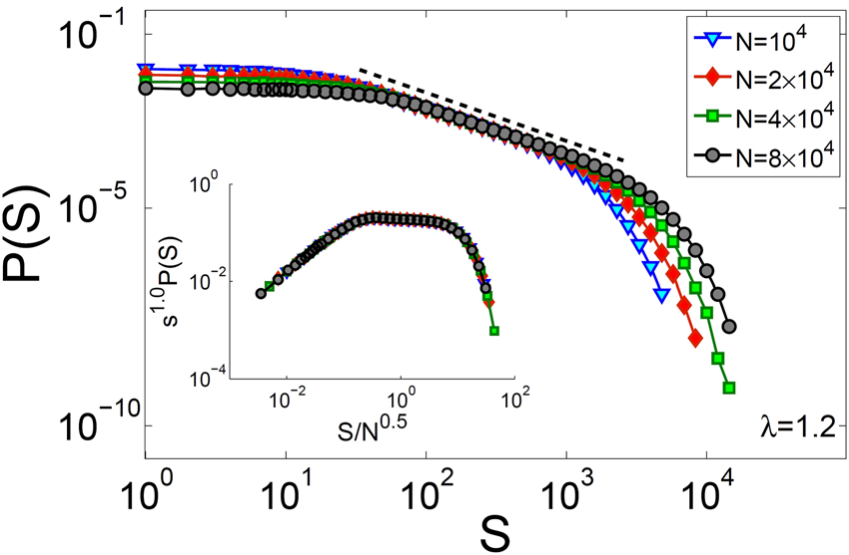
Main panel: probability distribution functions of avalanche sizes for systems with *λ* = 1.2 and different values of *N*. Inset panel: plots of rescaled data, collapsed into one universal curve with *τ*_*S*_ = 1.0 and *β*_*S*_ = 0.5.

Probability distribution functions of *S* are plotted in the main panel of Fig. 6 for systems with *λ* = 1.2 and different sizes. It is clearly seen that the plots exhibit a power-law region and a cutoff that increases by the system size. Inset panel of Fig. 6 shows collapse of the rescaled data of the main panel with exponents *τ*_*S*_ = 1.00 ± 0.01 and *β*_*S*_ = 0.50 ± 0.01. It must be noted that our numerical analysis show that different choices of *x*_*th*_ does not change the values of *τ*_*S*_ and *β*_*S*_, and we choose *x*_*th*_ = *x*_*c*_ where we have better statistics for avalanche distribution functions. Our numerical analysis for other values of 1 < *λ* < 2 indicate that the same values of *τ*_*S*_ = 1.00 ± 0.01 and *β*_*S*_ = 0.50 ± 0.01 are also obtained.

**Figure 7:**
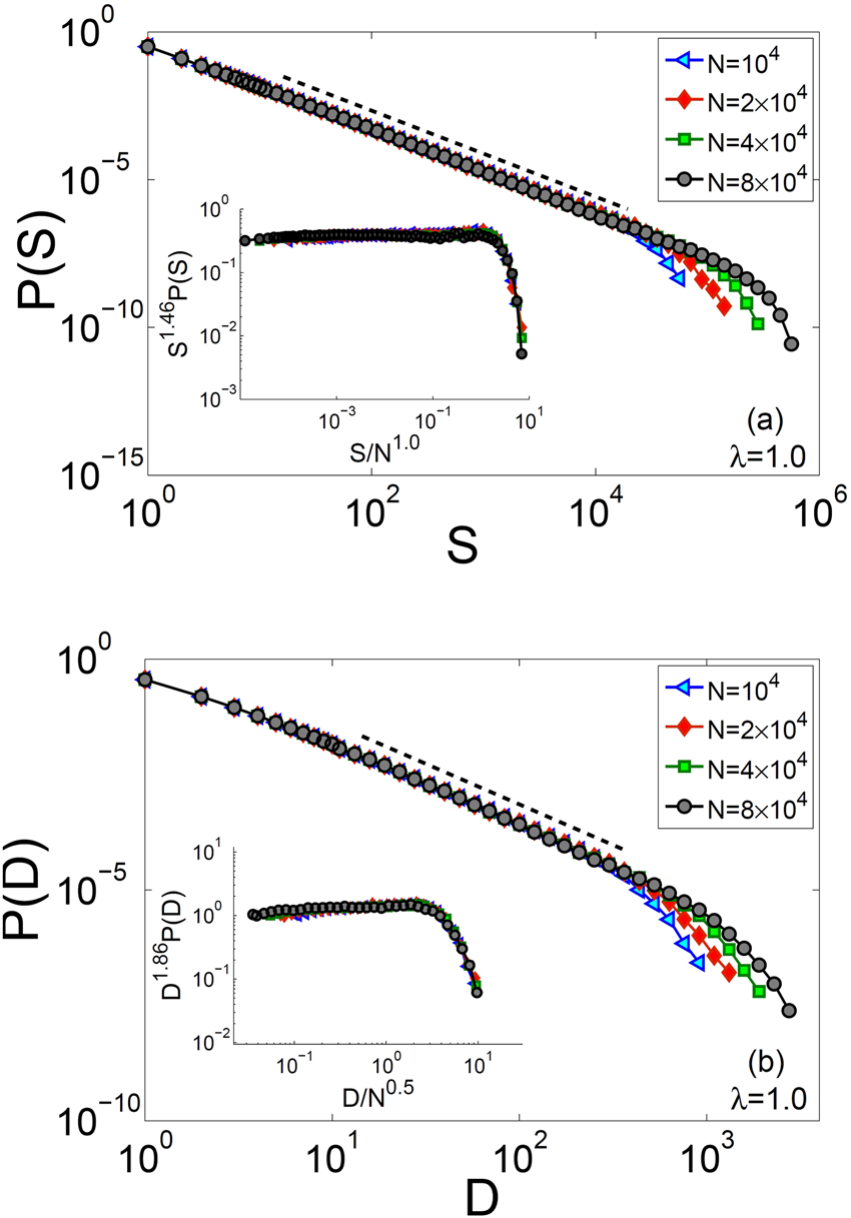
Main panels: probability distribution functions of (**a**) avalanche sizes and (**b**) avalanche durations for systems with *λ* = 1.0 and different values of *N*. Inset panel: plots of rescaled data, collapsed into one universal curve with *τ*_*S*_ = 1.46, *β*_*S*_ = 1.0, *τ*_*D*_ = 1.86, and *β*_*D*_ = 0.5.

However, the critical behavior obtained for *λ* = 1.0 is somewhat different. As shown in Fig. 7(a), we obtain *τ*_*S*_ = 1.46 ± 0.02 and *β*_*S*_ = 1.00 ± 0.03 which are indication of a different universality class for *λ* = 1.0, where the critical exponent is close to the critical branching process, i.e. *τ*_*S*_ = 3/2. More importantly, however, our study of finite-size scaling of avalanche sizes, in addition to *b*(*M*) → 1 presented earlier, provide firm evidence for critical behavior of the model in the range of 1 ≤ *λ* < 2. This could potentially provide an explanation to a wide range of criticality observed in neuronal systems, without any apparent tuning of parameters.

We have also calculated probability distribution functions of avalanche durations (*P*(*D*)). In Fig. 7(b), we have shown reslults for the *λ* = 1.0 case, where we obtain *τ*_*D*_ = 1.86 ± 0.02 and *β*_*D*_ = 0.50 ± 0.01 again close to the exponent *τ*_*D*_ = 2.0 of the critical branching process. However, the model exhibits an unusual scaling behavior for *D* in 1 < *λ* < 2 range. For system sizes we have been able to study (i.e up to *N* = 8 × 10^4^), probability distribution functions of avalanche durations *do* exhibit a power-law behavior. However, no appreciable increase is observed in the cutoff for the present system sizes. This behavior could possibly happen if the finite-size exponent *β*_*D*_ is so small that the cutoff function does not change considerably for the system sizes we have considered here.

**Figure 8:**
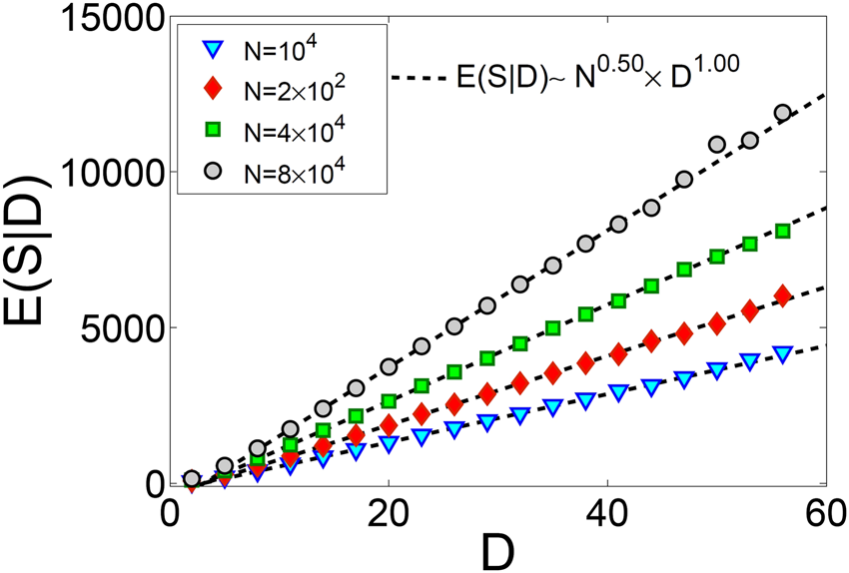
Plots of *E*(*S*|*D*) versus *D* for systems with *λ* = 1.2 and different values of *N*. Dashed lines are plots of *N* ^*α*^ × *D* ^*γ*^ fitted to the data with *α* = 0.5 and *γ* = 1.0.

To shed light on such a behavior, we consider a scaling anzats that relates the size and duration of avalanches as:

6 $$E(S|D)\sim {N}^{\alpha }{D}^{\gamma }$$

in which *E*(*S*|*D*) is the expectation value of *S* when *D* is given. As seen in Fig. 8, *E*(*S*|*D*) is a linear function of *D* for a given system size. Careful regression analysis shows that *α* = 0.50 ± 0.01 and *γ* = 1.00 ± 0.01. On the other hand, from the above numerical analysis, we know that the maximum value of avalanche sizes scales as ${S}_{max}\sim {N}^{{\beta }_{S}}$. Due to the linear relation between *E*(*S*|*D*) and *D* we can write *E*(*S*|*D*_*max*_) = *S*_*max*_. Using Eq. (6) we write ${S}_{max}\sim {N}^{\alpha }{D}_{max}$ which leads to ${D}_{max}\sim {N}^{{\beta }_{S}-\alpha }$ and therefore gives *β*_*D*_ = *β*_*S*_ − *α* = 0.00 ± 0.02. The fact that ${\beta }_{D}\approx 0$ indicates why we do not observe finite size scaling for avalanche durations despite the fact that we observe a power-law behavior for *P*(*D*) in a limited range of data. This is an interesting case whose full understanding requires further investigation with much larger system sizes than studied here.

### Sensitive dependence to perturbations

An interesting property of critical dynamical systems, such as self-organized critical models, is that short-term evolution of perturbations is a power-law function of time and the system exhibits power-law sensitivity to initial conditions^49^. In order to present another evidence for criticality of the system in the parameter regime 1 ≤ *λ* < *λ*_*p*_, we test this behavior in our model. In order to do that we consider a system in this parameter regime and run the simulation until the system reaches its critical” state. At this point we pause the simulation for a moment. We have the activity vector ${\bf{A}}(t)=\{{A}_{1}(t),{A}_{2}(t),{A}_{3}(t),\ldots ,{A}_{N}(t)\}$ in an *N* dimensional space and make a copy of the system **A**′. Then, we introduce a small perturbation to **A**′ by changing a few randomly chosen elements (${{\bf{A}}}_{i}^{^{\prime} }$) from one to zero or vice versa. The difference between two systems, which is a distance in the N dimensional space, is defined as the Hamming distance (*H*) between **A** and **A**′ which is

7 $$H(t)=\sum _{1}^{N}\,{({A}_{i}(t)-{A}_{i}^{^{\prime} }(t))}^{2}$$

**Figure 9:**
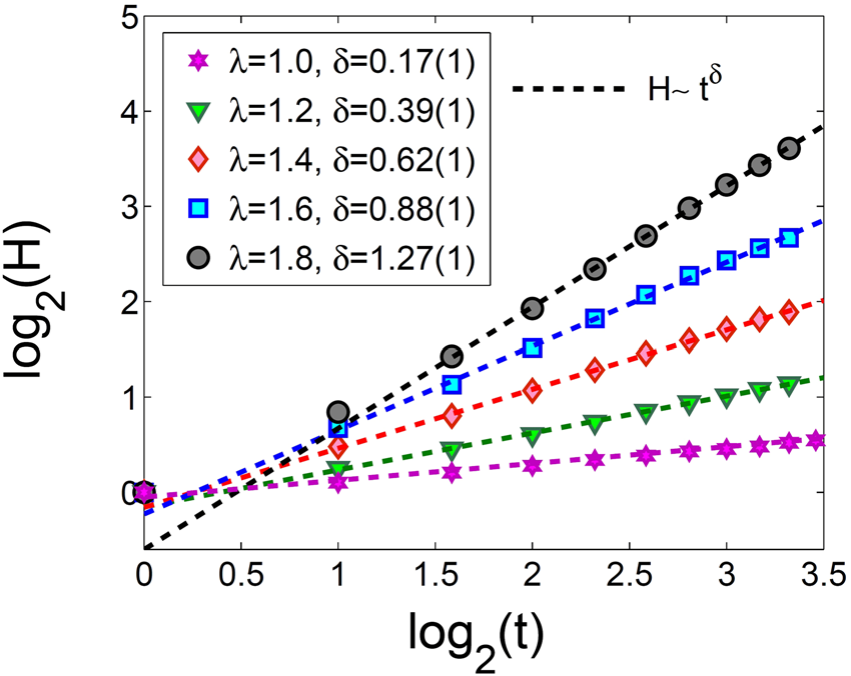
Short term evolution of the Hamming distance averaged over 2000 realizations for systems with one refractory time step, *N* = 40000, and different values of *λ* = 1.0, 1.2, 1.4, 1.6, 1.8. Linear fits are indications of power-law behavior.

Now, we can study the short-term evolution of *H*(*t*). It must be noted that we used the same random seed for simulating both systems. In order to have firm results we need to do an ensemble averaging, therefore, we have done the above process for 2000 realizations. Time evolution of the ensemble-averaged Hamming distance is plotted in Fig. 9 for systems with refractory period of one time step, *N* = 40000 and different values of *λ* = 1.0, 1.2, 1.4, 1.6, 1.8. It is observed that the system exhibits power-law sensitivity to initial conditions

8 $$H(t)\sim {t}^{\delta }$$

This provides yet another evidence for criticality of the system in the parameter range of 1 ≤ *λ* < *λ*_*p*_. Note that we have also included *λ*_*c*_ = 1.0 where criticality is well established. The exponent *δ* is an increasing function of *λ* indicating more chaotic behavior.

## Concluding Remarks

Motivated by the fact that scaling behavior is observed in systems composed of interacting excitable nodes, and that excitable nodes can exhibit refractory period, as in neuronal systems, we have studied a simple model of excitable nodes on a random directed graph in the presence of refractory period. The behavior of the model without refractory period has been well-studied previously.

We find that in the presence of refractory period the behavior of system undergoes dramatic changes and different dynamical regimes become accessible for the system. A sub-critical regime for *λ* < 1.0. A standard critical behavior with scaling and power-law behavior of avalanche sizes and durations, similar to the critical branching process, for *λ* = 1. An important regime with stable self-sustaining dynamics with interesting scaling behavior for 1 < *λ* < *λ*_*p*_ where activity dependent branching ratio goes to one in the thermodynamic limit and the system exhibits power-law statistics in avalanche distribution functions as well as power-law sensitivity to initial perturbations. The critical exponents associated with this new regime are distinct from those obtained from the standard critical point at *λ* = 1. Finally an oscillating regime with *λ* > *λ*_*p*_ where the dynamics oscillates between various well-defined states is observed. While our results highlights the importance of refractory period in networks of excitable nodes, it can also provide understanding for similar behavior observed in real neuronal systems. Branching ratios equal to one, as well as power law statistics in neuronal avalanches have been observed in a wide variety of real neuronal systems, with no apparent tuning of a parameter. The fact that our model exhibits similar behavior for a wide range of parameter (i.e. 1 ≤ *λ* < *λ*_*p*_) is the main result of our study. The more general case of longer refractory periods does not change our general conclusions and the details of such generalization appear in the Appendix.

In a recent work Larremore *et al*. have shown that inhibition causes ceaseless dynamics at or near the critical point ($\lambda \mathop{ < }\limits_{ \tilde {}}1$) in a similar model^43^. We, on the other hand, have also observed ceaseless stable dynamics but in a different parameter regime of *λ* > 1, when refractory period is included without inhibition. Both inhibition and refractory period are thought to decrease the level of activity in a system. However, they seem to lead to stable ceaseless dynamics in networks of excitable nodes. It would be interesting to study the effect of both these important properties simultaneously to see if larger parameter regime with stable dynamics and scaling (critical) behavior can be obtained.

It is generally believed that networks with small-world effect would exhibit critical behavior with exponents associated with critical branching process which correspond to mean-field exponents *τ*_*S*_ = 3/2 and *τ*_*D*_ = 2. Here, we have observed non-mean-field behavior with exponents which are significantly smaller than the critical branching process for a wide range of parameter in a small-world network of excitable nodes with refractory period. Of course, we have also obtained similar mean-field exponents for a particular value of *λ* = 1.

Although neuronal dynamics have been the main motivation of our work, other important dynamical processes such as epidemic spreading could also have relevance to our work as refractory period is thought to be important in such spreading processes as well.

## Numerical Details

Computer code simulations were developed in FORTRAN 90. In order to get good statistics for probability distribution functions of *x* as well as duration and size of avalanches, for each system size (*N* = 10^4^, 2 × 10^4^, 4 × 10^4^, 8 × 10^4^) we performed simulations for 20 different realization of networks and 10^6^ time steps for each network. Random networks were made by having every two node connected with a probability of *q* = 0.01.
